# Performance of Epoxy Resin Polymer as Self-Healing Cementitious Materials Agent in Mortar

**DOI:** 10.3390/ma14051255

**Published:** 2021-03-06

**Authors:** Ghasan Fahim Huseien, Abdul Rahman Mohd Sam, Iman Faridmehr, Mohammad Hajmohammadian Baghban

**Affiliations:** 1 UTM Construction Research Centre, Institute for Smart Infrastructure and Innovative Construction, School of Civil Engineering, Faculty of Engineering, Universiti Teknologi Malaysia, Skudai, Johor Bahru 81310, Malaysia; fhghassan@utm.my (G.F.H.); abdrahman@utm.my (A.R.M.S.); 2 Institute of Architecture and Construction, South Ural State University, Lenin Prospect 76, 454080 Chelyabinsk, Russia; s.k.k-co@live.com; 3 Department of Manufacturing and Civil Engineering, Norwegian University of Science and Technology (NTNU), 2815 Gjøvik, Norway

**Keywords:** self-healing concrete, epoxy resin polymer, strength development, healing efficiency, porosity, sustainability

## Abstract

This research investigated the application of epoxy resin polymer as a self-healing strategy for improving the mechanical and durability properties of cement-based mortar. The epoxy resin was added to the concrete mix at various levels (5, 10, 15, and 20% of cement weight), and the effectiveness of healing was evaluated by microstructural analysis, compressive strength, and non-destructive (ultrasonic pulse velocity) tests. Dry and wet-dry conditions were considered for curing, and for generating artificial cracks, specimens at different curing ages (1 and 6 months) were subjected to compressive testing (50 and 80% of specimen’s ultimate compressive strength). The results indicated that the mechanical properties in the specimen prepared by 10% epoxy resin and cured under wet-dry conditions was higher compared to other specimens. The degree of damage and healing efficiency index of this particular mix design were significantly affected by the healing duration and cracking age. An optimized artificial neural network (ANN) combined with a firefly algorithm was developed to estimate these indexes over the self-healing process. Overall, it was concluded that the epoxy resin polymer has high potential as a mechanical properties self-healing agent in cement-based mortar.

## 1. Introduction

Fissures and cracks are the main concerns for concrete due to its relatively low tensile strength, and this is one of the most severe problems affecting the durability and service life of concrete structures in the natural climate. At the early age of cementitious materials such as concrete, mortar, and paste, the loss of moisture from fresh mixture results in a reduction in volume. The restraint of mortar to volume changes caused initial shrinkage cracks. Initial shrinkage cracks in concrete normally occur in all building materials or components that are cement/lime-based such as concrete, mortar, masonry units, masonry and plaster, etc., and are one of the leading causes of cracking in structure. Initial shrinkage in concrete and mortar occurs during the construction of structural members due to drying out of moisture. The initial shrinkage of concrete is partly reversible if the moisture is maintained in concrete, but it becomes irreversible when concrete becomes dry [1,2]. During the concrete life cycle, several mechanical, chemical, and physical processes such as temperature gradients, plastic and autogenous shrinkage, external loading, and expansive reactions can create local stress and subsequently induce cracking [2,3,4]. Continuous cracks network significantly impaired durability and permeability of concrete, providing an easy path for the entrance of gases and liquids containing destructive substances that result in crack growth. Without proper and immediate treatment, such crack networks tend to spread and finally require costly treatment.

From a crack treatment perspective, conventional systems concentrate on the application of regular maintenance and scheming inspection. Nevertheless, in large-scale concrete structures, such conventional systems required a considerable amount of funds and labor. Furthermore, the treatment process somehow may be difficult or even impossible to implement where the affected structures are in service, i.e., large dams and tunnels. In these circumstances, self-healing concrete was proposed and has become increasingly attractive [5].

In concrete structures, self-healing of micro-cracks has already been introduced as a phenomenon that cracks networks could be automatically repaired by rehydration of insufficiently hydrated or un-hydrated cement particles in cracked areas. However, the previous research indicated that the crack width that can be healed in such technique is limited to a maximum of 0.1 mm, which is not enough for practical application [6]. In addition to autogenous healing, cracks may also be automatically closed using a specific healing agent in the cement matrix [7,8,9]. 

Self-healing properties can improve in concrete using different techniques including encapsulation of polymers, inclusion of fibers, and secondary hydration of un-hydrated cement [10]. The existing self-healing agents included cementitious materials, are classified broadly into the four groups such as the intrinsic healing [11], microbial healing [12], capsule-based healing [13], and vascular healing [14]. Another alternative self-healing material that has been attracted many researchers is bacterially produced calcium carbonate [4,15]. Gollapudi et al. [16] first proposed this technique in the mid-1990s to repair cracks using environmentally friendly processes. The technique involved incorporating ureolytic bacteria that facilitate enzymatic hydrolysis of precipitation of calcium carbonate and urea in the micro-crack regions.

The epoxy resin’s unique properties such as strong adhesion, high corrosion-resistance, and chemical durability made it attractive in the construction sector to produce modified concretes. Additionally, epoxy resin is advantageous as the admixture in the concretes because it can impart many emergent properties absent in the pure concretes. However, to produce the epoxy resin-modified mortar hardeners are essential [17,18,19]. According to Ariffin et al. [20,21], the inclusion of epoxy resin into the concretes imparts hardening properties when the alkaline substances are present. They developed the epoxy resin-modified mortars in the absence of any hardener. Ohama et al. [22] demonstrated that the epoxy resin-modified concretes and mortars in the absence of any hardener at (23 ± 3 °C) have a lesser hardening rate and strength performance than the one without epoxy resin. To overcome this drawback, the autoclave and steam curing techniques were followed to improve the early compressive strength (CS) developments of the epoxy resin-modified concretes [23,24]. Jo [25] observed that the characteristics of the epoxy resin-modified ordinary Portland cement (OPC)-based mortars in the absence of any hardener has relatively better properties than those with hardener. The observed increase in the strength performance was ascribed to the cross-linkages between the epoxy and the OPC network that could react with the Ca(OH)_2_ without any hardener [26].

The reaction ability between the epoxy resin and hydroxyl ions from the OPC hydration (Ca(OH)_2_) to produce the hardened epoxy motivated the researchers to apply epoxy resin as self-healing agent in concrete industries. It is established that the inclusion of the epoxy resin in the hydration process of the OPC matrix is responsible for enhancing the mechanical properties of the product. In the OPC hydration process, the produced hydroxyl ions are essential for the self-healing mechanism and C-(A)-S-H gels formation. In the bacteria-based self-healing concretes, the bacterial strain reacts with the oxygen to produce the calcite, thus precipitating and healing the concretes’ microcracks. In the epoxy resin-based self-healing concretes, similar mechanisms are involved, and also the epoxy resin can react with the hydroxyl groups, leading to the healing of the concrete cracks.

In this research, the epoxy resin without any hardener called diglycidyl ether of bisphenol A was adopted as the self-healing agent to produce a sustainable mortar for several construction applications with long life span and high durability. The mixes were designed with various ratios of the epoxy resin to OPC to assess the efficacy of the epoxy as the self-healing agent. The modified mortars were subjected to the dry and wet-dry conditions of curing to determine their improved properties such as flowability, CS development, porosity, degree of damage, and healing efficiency. The CS, ultrasonic pulse velocity (UPV), and SEM morphologies were recorded to determine the feasibility of implementing the epoxy resin as the self-healing agent in mortar manufacturing. In addition, an optimized artificial neural network (ANN) combined with a firefly algorithm was developed to estimate the degree of damage and healing efficiency output parameters over the self-healing process.

## 2. Materials and Experimental Program

### 2.1. Materials

**Figure 1:**
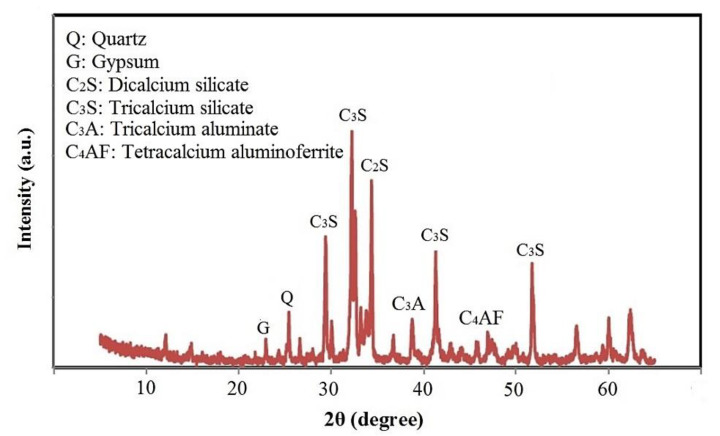
XRD patterns of the OPC.

In this study, the OPC was collected from the Holcim Cement Manufacturing Company (Johor Bahru, Malaysia) following ASTM C150 standard [27] to prepare the mortar. The X-ray fluorescence (XRF, HORIBA, Singapore, Singapore) spectra were analyzed to determine the main chemical compositions of the OPC by weight%, including the CaO (63.1%), SiO_2_ (20.1%), and Al_2_O_3_ (5.4%), where the total of loss on ignition (LOI) was 2.2 of the weight%. The mineral compounds of OPC, provided by the X-ray diffraction (XRD, Rigoku, Singapore, Singapore) pattern analyses, consist of Dicalcium Silicate (C_2_S), Tricalcium Silicate (C_3_S), tricalcium aluminate (C_3_A), and calcium aluminoferrite (C_4_AF) with the proportion of 9.3, 74.9, 6.7, and 9.1%, respectively, as shown in Figure 1. The low amorphous part of OPC was shown between 2θ 25 to 55 degree. The XRD pattern of OPC exhibits high pecks of C_2_S and C_3_S. These kind of elements will contribute to formulate the portlandite (Ca(OH)_2_) during the hydration process. Silicate minerals in non-crystalline or an amorphous state are highly reactive with Ca(OH)_2_ produced from the hydration of cement to form additional calcium silicate hydrate (C-S-H) gels. 

To develop self-healing properties in the mix design, epoxy resin was used without any hardener for the easy reaction with the hydroxyl ions and hardening. Therefore, the epoxy resin with high viscosity was selected where it was obtained using a Digital Brookfield Viscometer (Chongqing Gold Mechanical & Electrical Equipment Co., Ltd, Chongqing, China) while the epoxy equivalent, molecular weight, and its flash point were provided by the manufacturing company (Cemkrete Sdn. Bhd, Puchong, Malaysia). Table 1 summarizes the above-mentioned physical properties of the epoxy resin.

**Figure 2:**
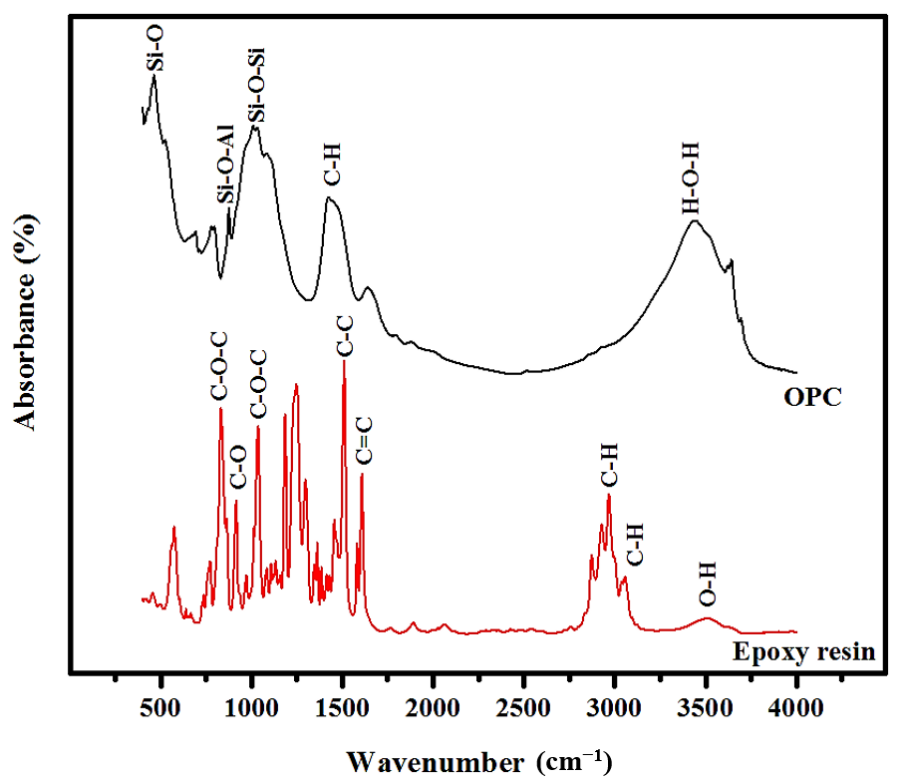
The FTIR spectra of the OPC and epoxy resin.

The Fourier transform infrared (FTIR, TA instruments, New Castle, DE, USA) of the epoxy resin, and OPC were considered to identify and quantify their bonding structures, compositions, functional groups, and hydration properties. Figure 2 demonstrates the main FTIR bands of the OPC and epoxy resin were in the range of 450 to 4000 wavenumber (cm^−1^). For the OPC, the bands at 462.9, 824, and 1033 cm^−1^ corresponded to the Si-O, Si-O-Al, and Si-O-Si bending. The previous literature reported that the broad band’s existence around 500–650 cm^−1^ was related to the glassy phase (short-ranged ordering of the network structures) of the silicates [28]. Meanwhile, the bands at 1426.7 and 3452 cm^−1^ were due to the C-H bending and H-O-H (water bands) stretching, respectively. The epoxy resin’s FTIR bands indicated the presence of different crosslinking mechanisms due to the chemical reactions between one or two kinds of monomers with different functional groups. The reactivity of the epoxies became completely different because of the alteration of the molecular structure. González et al. [29] showed the existence of highly reactive oxirane groups originated from the linkages between the aromatic rings and oxygen. Depending on the FTIR spectral analyses, the bands at 831, 916, and 1036 cm^−1^ were assigned to the C-O-C, C-O, and C-O-C stretching. The C-H and O-H stretching were observed in the range of 2871 to 3600 cm^−1^. In addition, the FTIR spectra of the epoxy resin without hardener appeared to be a valuable tool for both qualitative and quantitative analysis of these processes. It provided the specific information and reaction mechanisms to identify the epoxy resin components and the existence of different vibration bands. Moreover, the O-H band analysis rendered some information about the intermolecular interactions within the components of the epoxy resin.

**Figure 3:**
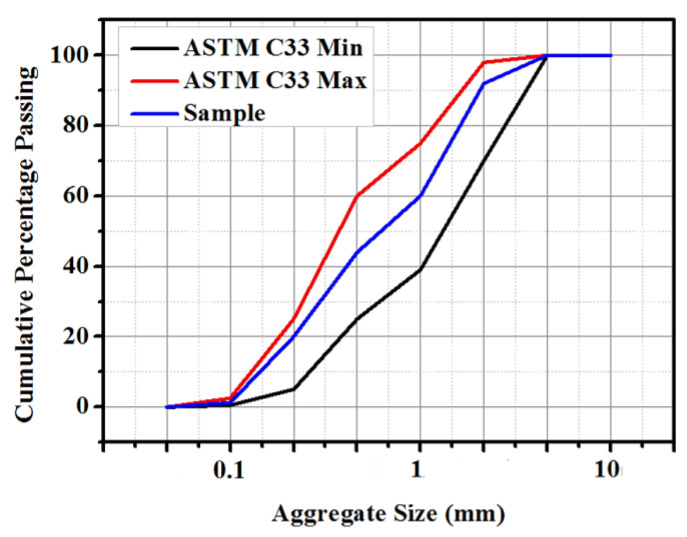
Particle size analysis of the fine aggregates.

The sieve analysis result of the river sand is shown in Figure 3. The retained percentage of the fine aggregates, the upper and lower limit, was selected according to the Standard Specification for Concrete Aggregates ASTM C33 [30]. In this research, the fine aggregate remains between the upper and lower limit to follow well-graded aggregate. River sand is known to have high silica content, 96% of the constituents, acting as filler in the epoxy-modified design mix. The percentage on fine aggregate remains constant in all mix designs. 

### 2.2. Mix Design, Casting, and Curing Condition

To evaluate epoxy resin’s adequacy as a self-healing agent, five batches of mix designs containing various epoxy resin levels were prepared in the laboratory, Table 2. The first batch was prepared without the epoxy resin and considered as the control sample. The control mixture was made by blending the OPC, fine aggregates, and water. In other batches, the epoxy resin to OPC content was varied from 5% to 20%. To obtain the desired strength properties of the mixes, the quality was controlled strictly during the materials preparation and mixing process by addressing the following criteria:

(1) Following to ASTM C1329 and ASTM C109 standards, the ratio of the cement to river sand and water to cement was fixed to 0.33 and 0.48, respectively for all mix designs;
(2) Municipal tap water was added to the concretes during the mixing and curing;
(3) During the preparation stage, a saturated surface dry condition of the river sand was adopted.

During the preparation stage, following ASTM C150 [31], first the river sand and OPC were blended together for 3 min, and then the epoxy resin was added to the mix and blended for another 2 min to get a homogenous blend. Subsequently, the water was added to the mix, and blending continued for another 3 min.

The workability of the mortars was measured using the flow table test. After the blend was mixed properly, the fresh mortar was poured onto the molds with two layers, where each layer was exposed to 15-s vibration to liberate the air and reduce the pores. The cubical samples with the dimension of 70 mm × 70 mm × 70 mm was used for the compressive strength (CS) test, cylindrical samples of dimension 75 mm × 150 mm was taken for the splitting tensile strength and the prism specimen of dimension 40 mm × 40 mm × 160 mm was prepared for the flexural strength test. Two types of curing conditions were applied on the obtained specimens including the dry and wet-dry. In the wet-dry state, the mortar was immersed in water for five days at (20 ± 3 °C). After five days, the specimens were taken out and left in the ambient condition (24 ± 3 °C) until the testing. In the dry curing, the mortar was left in the ambient state till testing. 

### 2.3. Fresh and Hardened Tests

The epoxy-modified mortars’ fresh characteristics were evaluated in terms of the workability using the flow table test in compliance with ASTM C230 [32]. The standard conical frustum with a diameter of 100 mm was used for the flow test, where the mortar was placed on the flow table and dropped 25 times within 15 s. As the mortar was dropped, it showed a spread out on the flow table, and the diameter of the spread mortar was recorded. This test was repeated three times to calculate the average. The setting times test of the cement pastes was carried out using the Vicat Apparatus (GA Precision Sdn Bhd, Shah Alam, Malaysia) according to the ASTM C191 [33]. In this test, the cement paste was placed into the conical mold of 80 mm diameter and 40 mm height in two layers where each layer tampered with 25 times with a rod. Then, the mortar paste was cured at room temperature, and at every interval of 15 min, the specimen was taken out and placed on the Vicat apparatus to measure the initial and final setting time.

The CS of specimens were measured at the age of 28 days using the universal testing machine (UTM, NL Scientific, Klang, Malaysia) with a 0.8 kN/s loading rate in compliance with ASTM C109 [34]. Similarly, the flexural and splitting tensile strength of epoxy-modified mortars were evaluated in compliance with the ASTM C78 [35] and ASTM C496 [36,37], respectively, after the curing age of 28 days. 

For the water absorption test, first three cubical epoxy-modified specimens were submerged inside water for 72 h (W_ts_) and after that, oven-dried for 24 h at 105 °C (W_td_). Each sample’s weight was measured and percentage of the water absorption (WA %) was calculated using the following equation in compliance with ASTM D 6532 [38].

(1) $$\mathrm{WA}\;=\;\left[\left(W_{\mathrm{ts}}\;-{\;W}_{\mathrm{td}}\right)/{\;W}_{\mathrm{td}}\right]\;\times \;100$$

W_ts_ and W_td_ (in kg) denote the weight of the treated mortar unit after the immersion in water and oven-dried. 

### 2.4. Self-Healing Evaluation Test

**Figure 4:**
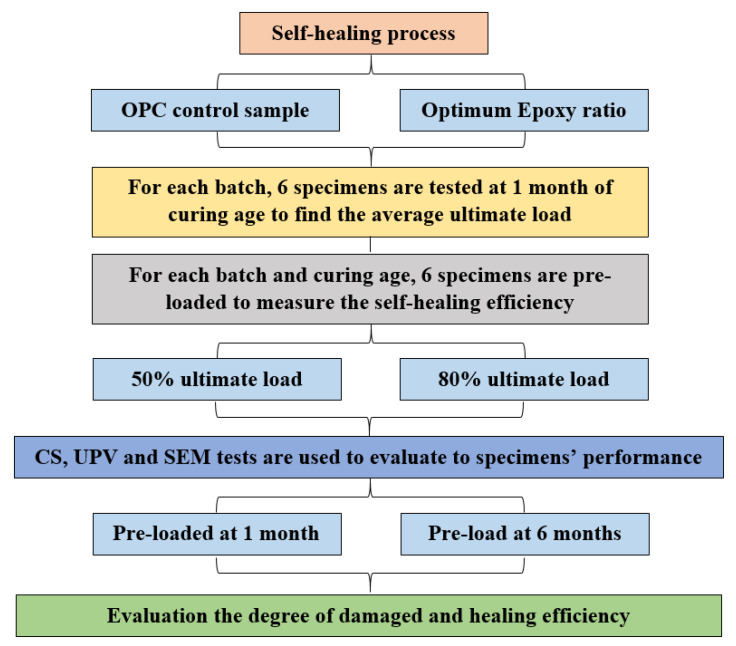
Self-healing evaluation procedure.

To evaluate the self-healing performance of the studied mortars, the artificial cracks were generated using a compression test machine (NL Scientific, Klang, Malaysia) (loading speed 0.8 kN/s) at the ages of 1 and 6 months. After generating artificial cracks, the pre-loaded and non-loaded (control samples) were left for curing at ambient temperature (27 ± 1.5 °C) under the relative humidity of 75%. The main reason to have a similar environment condition for all specimens was to ensure no external factor can affect the self-healing process. Figure 4 illustrated the self-healing evaluation procedure. Such an approach was intended to simulate the practical construction scenarios where the cracks often appear unexpectedly at a different age. At these proposed ages, the average of the developed CS, UPV, and the surface morphology (SEM, Hitachi, Ibaraki, Japan) of the specimens were recorded, and then the artificial cracks were generated on specimens by the pre-loading rate of 50 and 80% of their ultimate CS. At first, six specimens were selected from each batch (control sample and optimum ratio of epoxy content) and tested to find the ultimate load using a compression machine at one month of curing age. Then, 144 specimens were pre-loaded with 50% and 80% of the ultimate load to evaluate the crack width effect on epoxy risen efficiency as a self-healing agent. Subsequently, using structural morphology test, specimens were again investigated to ensure that the crack was properly generated inside the mortars, and then the specimens were cured at room temperature 27 ± 1.5 °C until the desired day of testing. Specimens were evaluated after 1, 2, 3, 5, and 11 months of pre-loading time, and the average value of six tested specimens was recorded for CS and UPV tests. The development CS and UPV of pre-loaded specimens were compared to non-loaded specimens at the same age. Meanwhile, the results of UPV were used to measure the degree of damaged using Equation (2). Equation (3) was also used to calculate the healing efficiency, and the development of compressive strength on non-loaded specimens. 

Suaris et al. [39] recommended that the UPV can characterize the development of the concrete damage. They proposed the following equation based on reducing the UPV, labeling the degree of damage.

(2) $$\mathrm{Degree}\;\mathrm{of}\;\mathrm{Damage}\;=\left[1-\;\left[\frac{V_{a}}{V_{b}}\right]\right]\;\times \;100$$

where V_a_ and V_b_ are the velocities after and before the peak loading (m/s) in the mortars.

The self-healing efficiency can also be determined using the following equation:

(3) $$\mathrm{Self}-\mathrm{healing}\;\mathrm{efficiency}\;=\;\left[\left({\mathrm{CS}}_{n}-{\mathrm{CS}}_{2}\right)-\left({\mathrm{CS}}_{m}-{\mathrm{CS}}_{1}\right)/{\mathrm{CS}}_{2}\right]\;\times \;100\;$$

where CS_1_ is the CS before the loading; CS_2_ is the CS after the loading; CS_n_ and CS_m_ are the CS of pre-loaded and non-loaded specimens at same curing age.

## 3. Results and Discussion 

### 3.1. Fresh Properties

**Figure 5:**
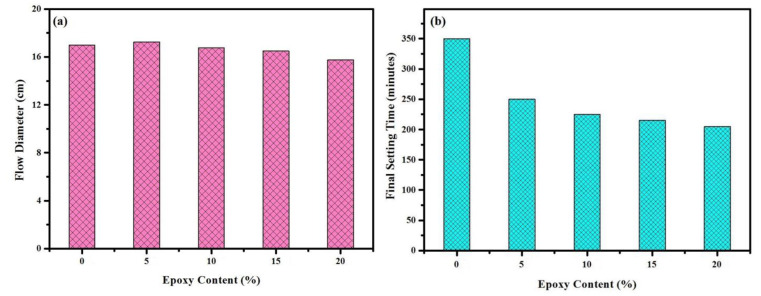
(**a**) Flow diameter and (**b**) final sitting time of all tested specimens.

The effects of the epoxy resin on the workability of the prepared mortars were measured using setting time and the flow table test. Figure 5 illustrates the values of the measured flow diameter and final sitting time for all tested specimens containing different percentages of epoxy resin. The results indicated that the flow diameters of the fresh mortar were in the range of 169 ± 3 mm, and by increasing the epoxy resin content, the flow diameter tended to decrease as a result of its high viscosity (Figure 5a). Similarly, the final sitting time was significantly decreased in a modified mortar containing epoxy resin compared to a conventional one (Figure 5b). For example, the sitting time was decreased by around 70% in the specimen containing 20% epoxy resin compared to the control specimen. Such a phenomenon can explain by the fact that the mortar mixes became very sticky with the rising levels of the epoxy resin; shorten the setting time of the modified mortar mixture. This observation was consistent with the findings of Khalid et al. [40].

### 3.2. Mechanical Properties 

**Figure 6:**
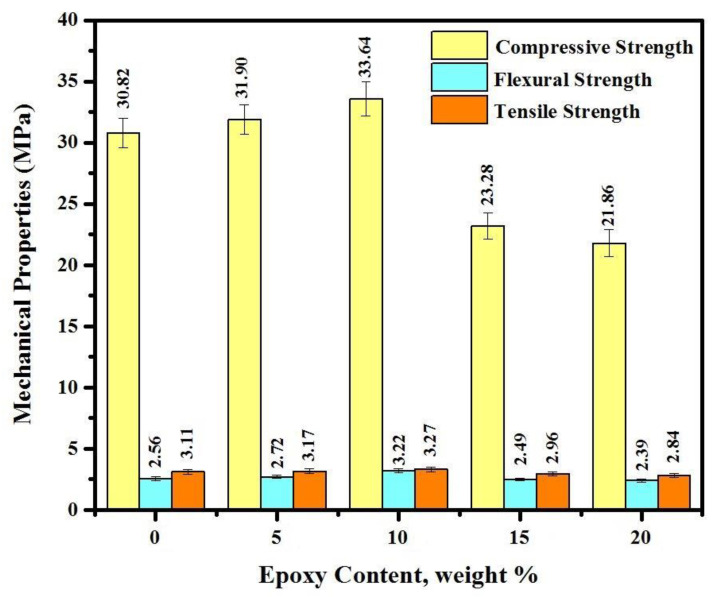
Mechanical properties of all tested specimens.

Figure 6 shows the mechanical properties of epoxy resin modified mortar at 28 days of dry curing age. Three specimens were tested for each mixture and average value considered for compressive strength (CS), Flexural strength (FS) and splitting tensile strength (TS). The results indicate that the mechanical properties were improved by an average of 3 and 9% with increasing epoxy resin from 0 to 5 and 0 to 10%, respectively. The highest mechanical properties were achieved by a specimen containing 10% epoxy resin with CS of 33.6 MPa, FS of 2.8 MPa, and splitting TS of 3.3 MPa with standard deviation (SDV) ±1.6, ±0.12 and ±0.22, respectively. Improving the mechanical properties in specimens with 5 and 10% of epoxy resin attributed to the presence of OH^−^ ions from the hydration of Ca(OH)_2_. Meanwhile, the results confirm that the mechanical properties tend to decrease by increasing epoxy resin content to from 10 to 15 and 20% significantly. For instance, the CS declined by 54% by increasing the epoxy resin from 10 to 20%. The previous literature indicated that by increasing the epoxy content by more than 10% in the mix design, the residual unhardened epoxy within the mortar matrix might have interrupted the hydration and polymerization processes [41]. It was also acknowledged that the mechanical properties in epoxy resin modified specimens were increased by an average of 8% under wet-dry curing condition regardless of epoxy resin content.

**Figure 7:**
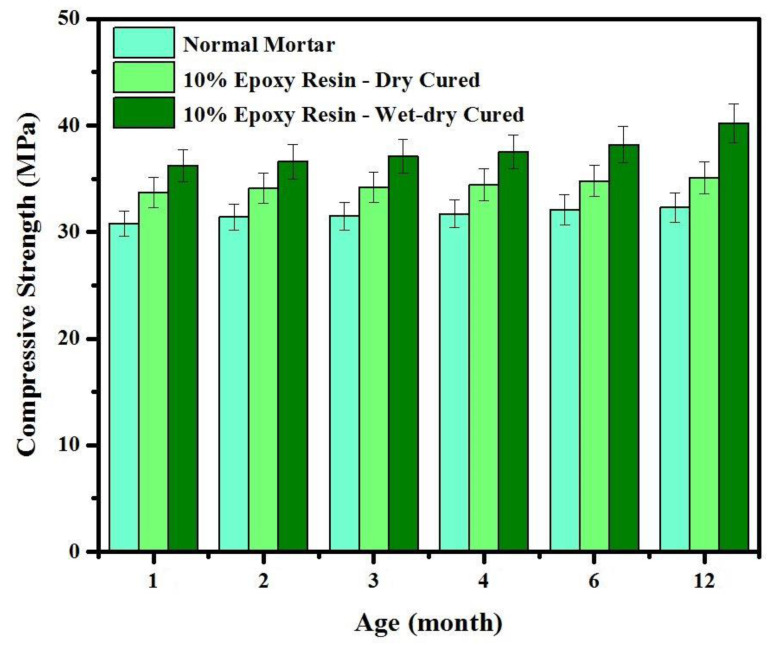
Effects of curing age on compressive strength development.

Figure 7 shows the development of CS in epoxy resin modified mortars at different ages using dry and wet-dry curing conditions. The average values of three readings were adopted for each test with SDV between ±1.4 to ±1.8. It is clear that the CS was increased for all specimens by increasing the curing age. Nevertheless, the increase rate was substantially higher in specimen containing 10% epoxy resin cured under wet-dry curing conditions where the CS was increased from 36 MPa at the curing age of 28 days to around 40 MPa at the curing age of 360 days. Such improvement attributed to the OH^−^ content that improve the hydration process and increasing the C-S-H gel product [42]. 

**Figure 8:**
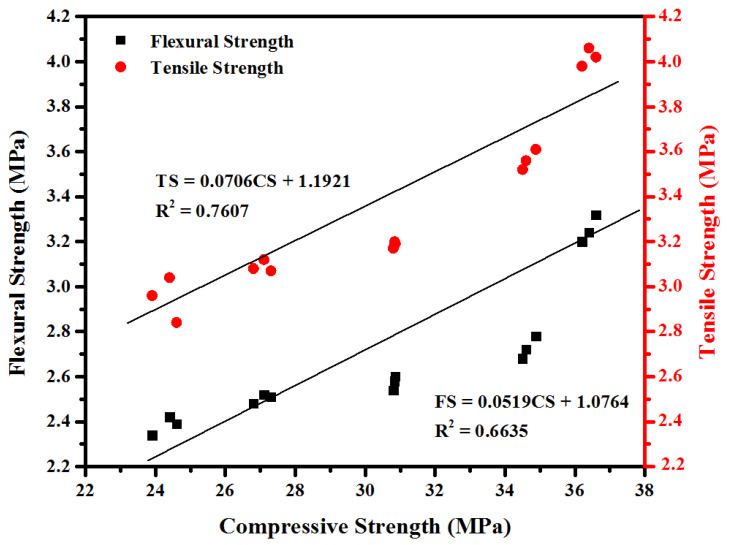
Correlation between mechanical properties of epoxy resin modified specimens.

Using multi-linear regression analysis, Figure 8 shows the correlation among mechanical properties of epoxy resin modified specimens after 28 days of curing. The ACI [43] proposed the following correlation for mechanical properties of concrete.

(4) $$\mathrm{TS}=0.55\sqrt{\mathrm{CS}}$$

(5) $$\mathrm{FS}=0.62\sqrt{\mathrm{CS}}$$

The proposed empirical equations by multi-linear regression analysis are in good agreement with ACI equations.

### 3.3. Microstructural Analysis 

**Figure 9:**
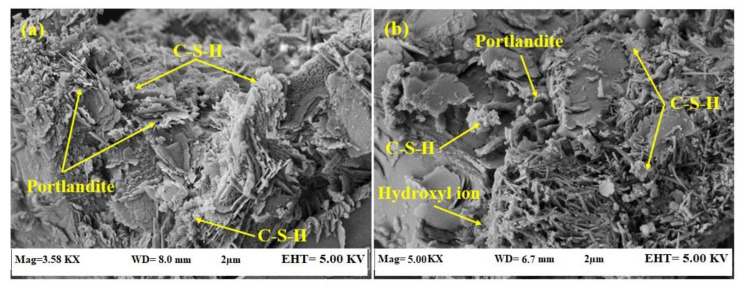
SEM micrographs of the (**a**) control and (**b**) 10% epoxy based mortar.

Figure 9 illustrates the SEM image of the control and 10% epoxy resin modified specimens. The results (Figure 9a) indicated the production of C-(A)-S-H gels and Ca(OH)_2_ in the control specimen that are primary products of the cement hydration. Figure 9b shows that the specimen prepared with 10% epoxy resin contain hydroxyl–ion–epoxy resin that led to the microstructure’s improvement, reduced the porosity, and providing high strength performance compared to the control specimen. In the cement hydration process, the Ca(OH)_2_ was generated, and it was consumed partially over C-(A)-S-H production. In the 10% epoxy resin modified specimen, the remaining Ca(OH)_2_ was reacted with the unhardened epoxy resin, creating strong bonds between the hydroxyl ions and epoxy to increase the CS. The continuous hydration reaction in the epoxy-modified mortar initiated the polymerization reactions. These reactions further strengthened the bonds and filled the pores within the mortar. Consequently, the improved mechanical behavior was achieved in the epoxy-modified mortars, particularly the one containing 10% of the epoxy resin.

**Figure 10:**
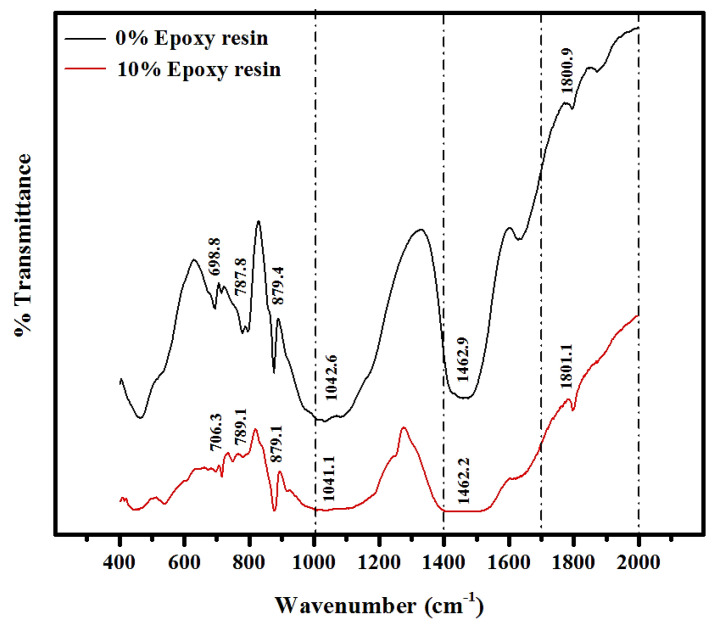
FTIR spectra analysis of the OPC and 10% epoxy resin modified mortar.

Figure 10 shows the FTIR spectra of the normal and 10% epoxy resin modified mortars, which consisted of various chemical functional groups corresponding to the bonding vibration modes. The results indicated that the epoxy resin modified specimen has a higher Ca(OH)_2_ absorption due to residual hydroxyl ions used in the epoxy resin reaction. The FTIR spectrum indicated the Si-O, Al-O, and C-H reaction regimes in the mortar metric. The Si-O and Al-O bond vibration energy levels were increased from 698.8 and 787.8 cm^−1^ to 706.3 and 789.1 cm^−1^ with a corresponding rise in the epoxy resin level from 0 to 10%. Increasing demand of the OH^−^ ion and its consumption in the production of the hardened epoxy (hydroxyl-ions-epoxy resin) affected the total amount of Ca (OH)_2,_ which further reduced the consumption of the silica-aluminum in the hydration process. The increase in the bond vibration energy 875 to 1045 cm^−1^ was attributed to the generation of the high quantity of Al(OH)_4_, C-S-H, and C-A-S-H gels apart from the hydroxyl-ions-epoxy resin, improving the mechanical performance. The distortion of the bonds in the range of 1100 and 1800 cm^−1^ might be relevant to the H-O-H’s bending vibrations in the H_2_O molecules, whereas the C-C ring of the phenol group showed stretching in the epoxy-modified specimen. The addition of 10% of the epoxy resin activated some interactions with the hydroxyl ions, and the reaction of the Ca(OH)_2_ with the epoxy resin caused the polymerization processes. The amount of Ca(OH)_2_ was found to decrease as the polymer became reactive.

### 3.4. Water Absorption (WA)

**Figure 11:**
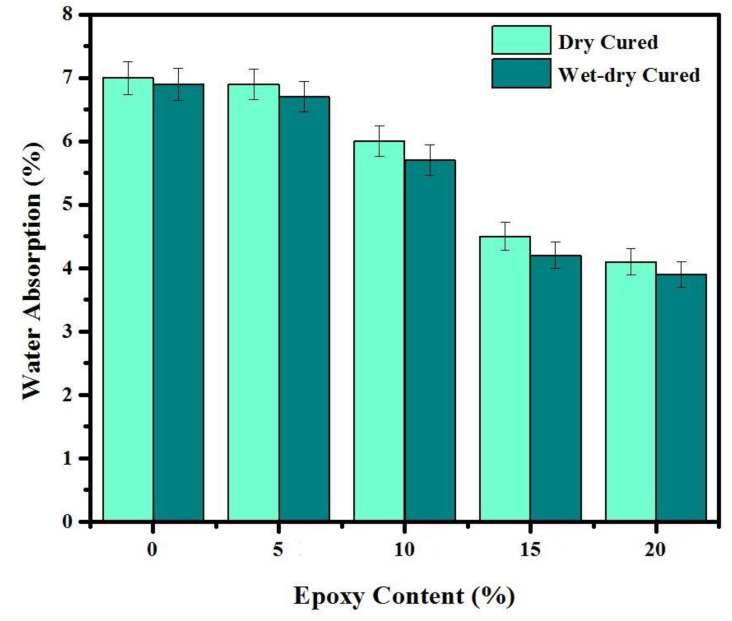
Effect of epoxy resin content and curing condition on water absorption.

Figure 11 shows the effect of various ratios of epoxy resin on water absorption (WA). The average values of three readings were adopted for each mixture. The results indicated that the water absorption inversely proportional with the content of epoxy resin content. The WA was declined by around 70% in specimen containing 20% epoxy resin compared to the control specimen. The specimens cured under dry conditions absorb water by average 6% higher compared to wet-dry cure conditions due to the reaction of the OH^−^ with the unhardened epoxy resin in the modified mortars. 

### 3.5. Self-healing Evaluation

**Figure 12:**
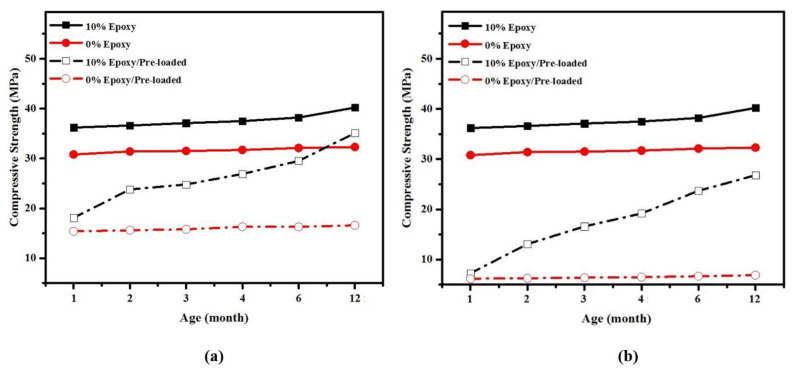
Self-healing efficiency of epoxy resin on CS development of pre-loaded specimens (**a**) 50% (**b**) 80%.

**Figure 13:**
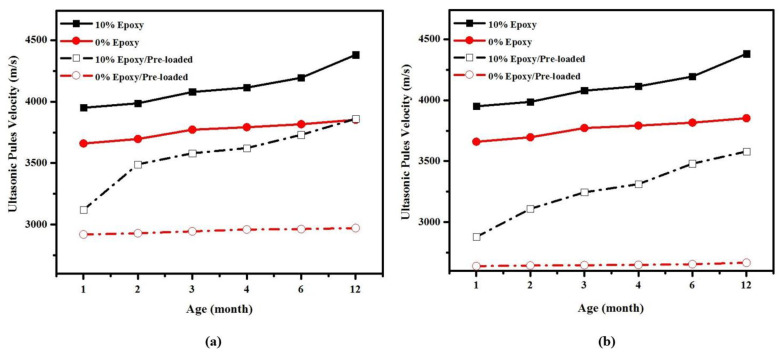
Self-healing efficiency of epoxy resin on UPV reading of pre-loaded specimens (**a**) 50% (**b**) 80%.

The self-healing efficiency of epoxy resin on CS and pulse velocity under 50 and 80% pre-loading is presented in Figure 12 and Figure 13 for control specimen and specimen containing10% of epoxy resin, cured under wet-dry condition. In both specimens, pre-loading was applied at one month of curing age. The self-healing function was evaluated by pre-loading mortars by 50% and 80% of maximum load and the specimens were checked by using non-destructive test method. Two types of mixtures were adopted to evaluate the self-healing function; the first mixture was prepared with 0% epoxy resin and considered a control sample. The second mixture was prepared with 10% of epoxy resin and cured a wet-dry environment. 

For the specimen adopted as a control sample, the strength was dropped from 30.8 MPa to 15.4 MPa after pre-loading 50% of the maximum load (Figure 12a). However, the specimens showed a slow rate of CS development, and the strength recorded 15.6, 15.9, 16.3, 16.4, and 16.8 MPa after 1, 2, 3, 5, and 11 months of pre-loading age, respectively. A similar trend was also observed with control specimens which were exposed to 80% pre-loading, and the very slow rate of strength development was recorded; the average CS after 11 months of pre-loaded date (12 month of curing age) presented 6.9 MPa compared to 6.2 MPa at the initial pre-loading (Figure 12b). Unlike, the specimens containing 10% epoxy resin presented excellent performance compared to the control sample. After 50% of pre-loading and reduced the strength from 36.2 MPa to 18.1 MPa, the compressive strength of cured specimens was developed and recorded 23.8, 24.8, 26.9, 29.5, and 35.1 MPa after 1, 2, 3, 5 and 11 months of pre-loading date, respectively. Specimens with 80% pre-loaded showed lower performance than that pre-loaded with 50% of maximum load, and the CS was recorded 13.1, 16.6, 19.2, 23.7, and 26.8 MPa for the same periods of pre-loading. Meanwhile, the OPC did not show a noticeable improvement after 5 or 11 months of pre-loading date. The results shown that the efficiency of epoxy resin as self-healing agent was highly influenced by the crack width. Specimens pro-loaded with 80% of ultimate load displayed lower performance in the healing process than that pre-loaded with 50% of the ultimate load. The ability of the mortars in regaining the initial CS clearly indicated the epoxy stimulated self-healing action. It can be asserted that the use of the epoxy resin in the absence of any hardener as the self-healing mediator is beneficial for the production of the maintenance-free mortar, thereby contributing to sustainable development in the building sectors. 

The results indicate that the healing efficiency on CS and UPV is much higher for specimens containing 10% epoxy resin than conventional mortar. The CS in epoxy-modified specimen significantly improved, from 18.1 MPa after artificial crack generation using 50% pre-load to around 35 MPa over 11 months of healing duration. Almost the same trend was observed for pulse velocity, where the velocity in epoxy-modified specimens was increased by an average of 23.7% after spending 11 months of healing duration. This is an indication that the structure of the specimen is dense with fewer pores and cracks. The results also acknowledged that the CS and pulse velocity in epoxy resin modified specimen substantially recovered after generation artificial cracks by 80% pre-load and reached initial condition after spending 11 months of healing duration. On the other hand, a low rate of enhancement was observed by conventional specimen after 11 months of pre-loading age (12 months of curing age), which significantly affects its durability performance. 

**Figure 14:**
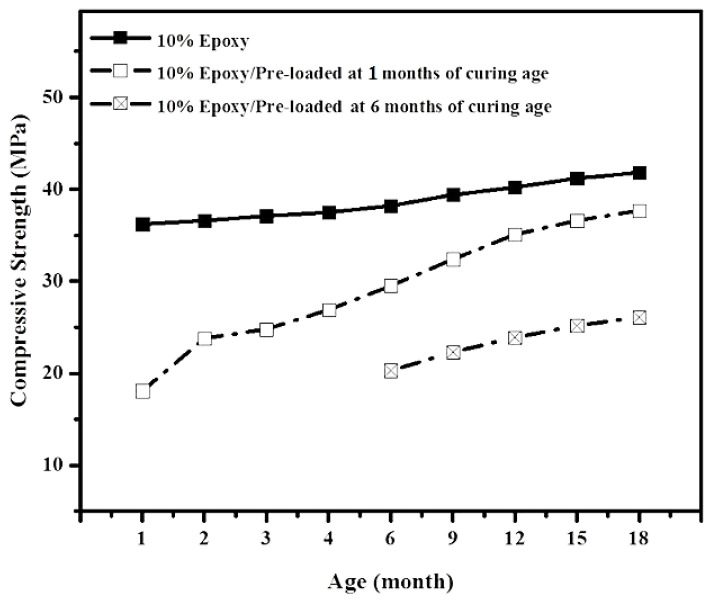
Effect of artificial cracks age on CS and healing efficiency development.

Figure 14 shows the effects of artificial crack generation age on CS healing efficiency of epoxy-modified mortar. The results indicate that the CS was recovered to its initial condition once the cracks were generated at the early age of curing, indicating epoxy resin’s superior efficiency to healing the cracks. Figure 14 shows that the CS was recovered from 18 MPa after generating by 50% pre-load at the curing age of 1 month to over 35 MPa after 11 months of healing duration, indicating that cracks were completely healed by 70%. On the other hand, once the artificial cracks were generated at 6 months of curing age, the CS only recovered by 20%, from around 19 MPa after generation of artificial cracks to 26 MPa after 11 months healing duration. Overall, it was concluded that there is a direct relationship between artificial crack generation age and self-healing efficiency, where a higher healing efficiency was achieved at a younger crack age. Figure 14 also confirmed that after the generation of artificial cracks, the control samples without epoxy did not recover and not shown any gain in compressive strength. 

**Figure 15:**
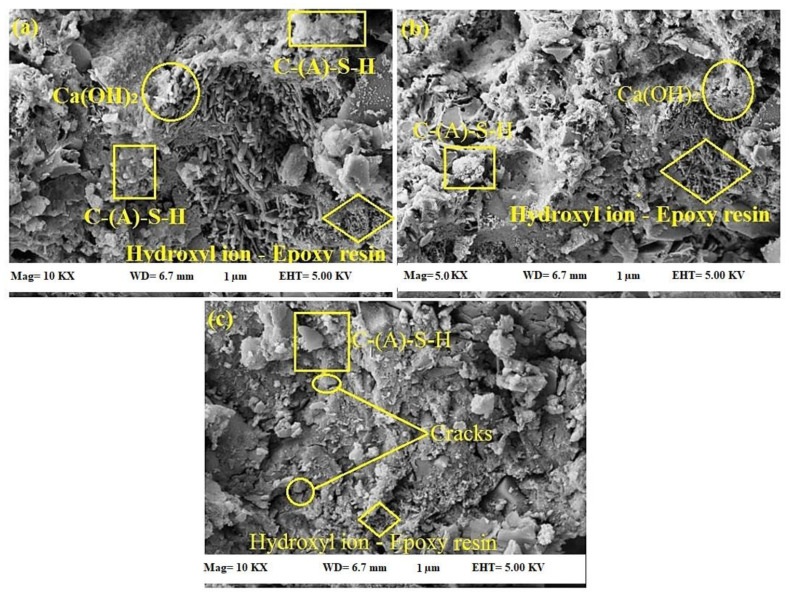
SEM morphology of self-healing mechanism of epoxy-modified specimen after artificial cracks generation (**a**) 28 days (**b**) 180 days (**c**) 365 days of curing age.

Figure 15 shows the SEM morphology of the self-healing mechanism of epoxy-modified specimens where artificial cracks were generated at 28, 180, and 365 days of curing age. The healing efficiency depends on the age of artificial cracks generation and the amount of the unhardened epoxy resin and Ca(OH)_2_ available in the specimen’s matrix. After the generation of artificial cracks, the unhardened epoxy started reacting with the OH^−^ from calcium hydroxide that becomes hardened and filling the cracks. Figure 15a shows the epoxy-modified specimen’s self-healing mechanism where artificial cracks were generated at 28 days of curing age. This figure shows that the produced cracks were occupied by the reaction products of the epoxy resin and OH^−^. Additionally, the high quantity of the C-S-H gel and Ca (OH)_2_ were observed from SEM results, where they are the main components associated with strength enhancement and healing the cracks.

On the other hand, the SEM morphology shows once the artificial cracks were generated at the later age, Figure 15b,c, a lower amount of C-S-H gel, Ca (OH)_2_, and hardened epoxy resin were produced, negatively affected the healing efficiency. In the self-healing mechanism, the OH^−^ generated in the hydration process was significant for producing the C-(A)-S-H gels and self-healing system. The hydroxyl ion was needed for the occurrences of the self-healing action. In the bacteria-activated self-healing concretes, the bacterial strain reacts with the oxygen to generate the calcite, further precipitates to heal the cracks. Using a similar mechanism, the epoxy-modified mortars were prepared where the epoxy resin reacted with the OH^−^ that enabled the hardening and healing of the cracks. Half of the epoxy resin was hardened, and the remaining one reacted as the healing agent at a later age. The reaction pathway followed the following mechanism:OH^−^ (From cement hydration) + Unhardened epoxy resin → Hardened epoxy resin 
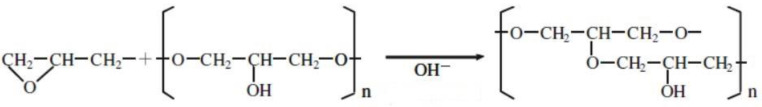
(6)

Table 3 shows the degree of damage and healing efficiency as a measure to evaluate the adequacy of epoxy resin to healing the artificial crakes. Similar to CS and UPV, the degree of damage and healing efficiency was a function of the generation age of artificial cracks. A superior performance had achieved by epoxy-modified specimen once the artificial cracks were generated at an early age (1 month) where the degree of damage and healing efficiency surpasses 2.3 and 71.8%, respectively. On the other hand, once the artificial cracks were generated at the later curing age of 6 months, the healing efficiency was declined by 70%. The degree of damage also was recorded 17.8 (from the initial damage of 26%) in these specimens after spending 11 months of healing duration. 

## 4. Developing ANN to Estimate Degree of Damage and Healing Efficiency

This section develops an ANN combined with a metaheuristic algorithm to estimate the degree of damage and healing efficiency of studied epoxy-modified specimens. 

The multilayer feed-forward network provides a reliable feature for the ANN structure and, therefore, was used in this research. The multilayer feed-forward network comprises three individual layers: the input layer, where the data are defined to the model; the hidden layer/s, where the input data are processed; and finally, the output layer, where the results of the feed-forward ANN are produced. Each layer contains a group of nodes referred to as neurons that are connected to the proceeding layer. The neurons in hidden and output layers consist of three components; weights, biases, and an activation function that can be continuous, linear, or nonlinear. Standard activation functions include nonlinear sigmoid functions (logsig, tansig) and linear functions (poslin, purelin) [44]. Once the architecture of a feed-forward ANN (number of layers, number of neurons in each layer, activation function for each layer) is selected, the weight and bias levels should be adjusted using training algorithms. One of the most reliable ANN training algorithms is the backpropagation (BP) algorithm, which distributes the network error to arrive at the best fit or minimum error [45,46].

### 4.1. Firefly Optimization Algorithm (FOA)

The fireflies, also known as lightning bugs, are nocturnal, luminous beetles. Several researchers have studied the behavior of this creature in nature [47]. The nature-inspired firefly algorithm is a metaheuristic algorithm proposed by Xin-She Yang et al. [48] and stimulated by its flashing behavior. In the classic firefly optimization algorithm, two fundamental aspects need to be clarified: the source light and attractiveness. The intensity of light I is referred to as an absolute measure of emitted light by the firefly, while the attractiveness β is the measure of light seen by the other fireflies. The intensity of light is defined using following equation:

(7) $$I\left(r\right)=I_{0}e^{-{\gamma r}^{2}}$$

where I_0_ represents the intensity of the source light and γ is the absorption of light by approximating the constant coefficient. The attractiveness β define using the following equation.

(8) $$\beta =\beta_{0}e^{-{\gamma r}^{2}}$$

where β_0_ is the attractiveness at the Euclidean distance γ is define using the following equation between two fireflies s_i_ and s_j_, and is equal to 0:

(9) $$\gamma_{\mathrm{ij}}=\|s_{i}-s_{j}\|\sqrt{\overset{k=n}{\underset{k=1}{\sum }}{\left(s_{\mathrm{ik}}-s_{\mathrm{jk}}\right)}^{2}}$$

According to Equations (7) and (8), the firefly algorithm has two asymptotic behaviors. If γ→0, the attractiveness tend to be constant (β = β_0_), and if γ→∞ the firefly movement follows at a random walk. The firefly algorithm is being attractive in civil engineering and material sciences. Bui et al. [49] used the firefly algorithm to predict the compressive and tensile strength of high-performance concrete. Sheikholeslami et al. [50] developed an improved firefly algorithm to optimize reinforced concrete retaining walls. Nigdeli et al. [51] developed a firefly algorithm to optimize reinforced concrete footings.

### 4.2. Generation of Training and Testing Data Sets

To train and develop a reliable ANN, epoxy-modified design mixes’ mechanical and material properties were taken into account based on input variables, Table 4. 

**Figure 16:**
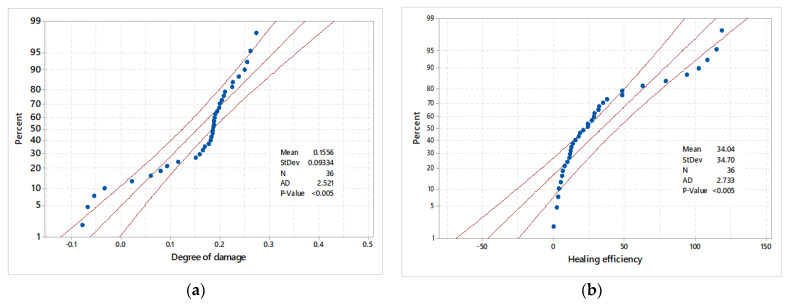
Probability plot for output variables (**a**) degree of damage and (**b**) healing efficiency.

Since the behavior and number of input data should be statistically evaluated against the output data, Figure 16 shows the probability plot of output data.

The ANN used in this study is a new feed-forward model. Eighty percent of input data, out of 36 samples, were used for training, and the remaining 20% were considered for testing the network. According to the characteristics of the available input data and the number of outputs, a two-layer ANN was proposed in the initial attempt, and its adequacy was evaluated by using several measures. Therefore, the trial and error method is used to obtain the ideal architecture, the architecture that best reflects the characteristics of the laboratory data. In this research, an innovative method for calculating the number of neurons in hidden layers was taken into account, as shown in the following Equation

(10) $$N_{H}\leq 2N_{I}+1$$

where N_H_ is the number of neurons in the hidden layers and N_I_ is the number of input variables. 

Since the number of effective input variables is 6, the empirical equation shows that the number of neurons in hidden layers can be less than 13. Therefore, several networks with different topologies, with a maximum of two hidden layers and a maximum of 13 neurons, were trained and studied in this study. The hyperbolic tangent stimulation function and Levenberg—Marquardt training algorithm were used in all networks. The statistical indices used to evaluate the performance of different topologies are Root Mean Squared Error (RMSE), Average Absolute Error (AAE), Model Efficiency (EF), and Variance Account Factor (VAF) that are defined as follows.

(11) $$\mathrm{MSE}=\frac{1}{n}\overset{n}{\underset{i=1}{\sum }}{\left(P_{i}-O_{i}\right)}^{2}$$

(12) $$\mathrm{ME}=\frac{1}{n}\overset{n}{\underset{i=1}{\sum }}\left(P_{i}-O_{i}\right)$$

(13) $$\mathrm{MAE}=\frac{1}{n}\overset{n}{\underset{i=1}{\sum }}\left|P_{i}-O_{i}\right|$$

(14) $$\mathrm{RMSE}={\left[\frac{1}{n}\overset{n}{\underset{i=1}{\sum }}{\left(P_{i}-O_{i}\right)}^{2}\right]}^{\frac{1}{2}}$$

After examining different topologies, it was found that the network with 6-6-4-2 topology has the lowest value of error in RMSE, AAE, EF, VAF and the highest value of R^2^ to estimate the two output parameters as shown in Table 5. It is necessary to mention that the error criteria for training and testing the data are calculated in the main range of variables and not in the normal range. 

**Figure 17:**
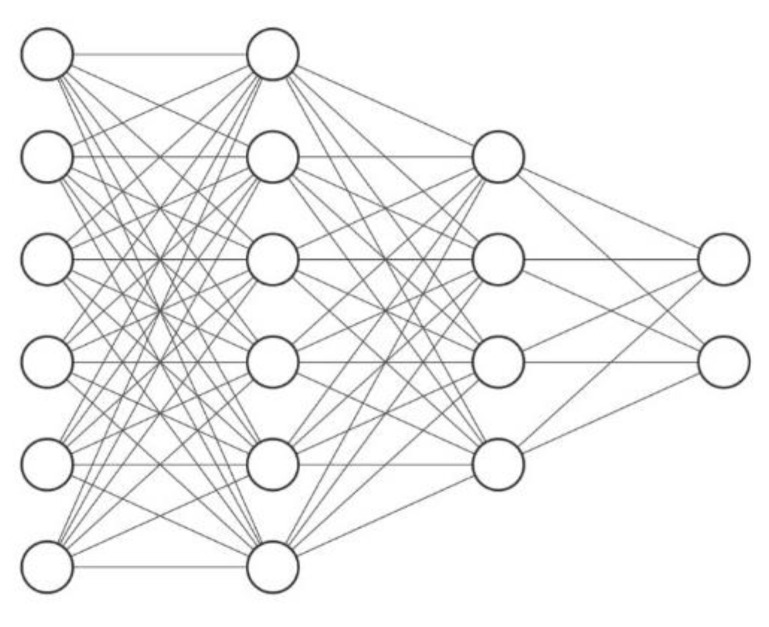
The topology of a feed-forward with two hidden (6-6-4-2).

Figure 17 shows the topology of a feed-forward network with two hidden layers, six input variables (neurons), and two output parameters.

To optimize the ANN’s weights and biases, the FOA has been used to provide the least prediction error for trained structure. The properties of the FOA parameters are shown in Table 6.

### 4.3. Results 

**Figure 18:**
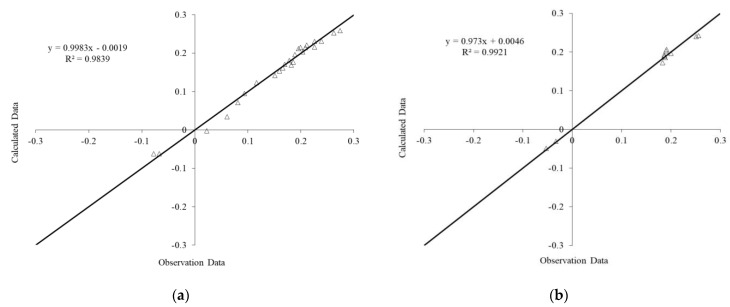
Predicted versus Experimental values of degree of damage output using (**a**) training data, (**b**) testing data.

**Figure 19:**
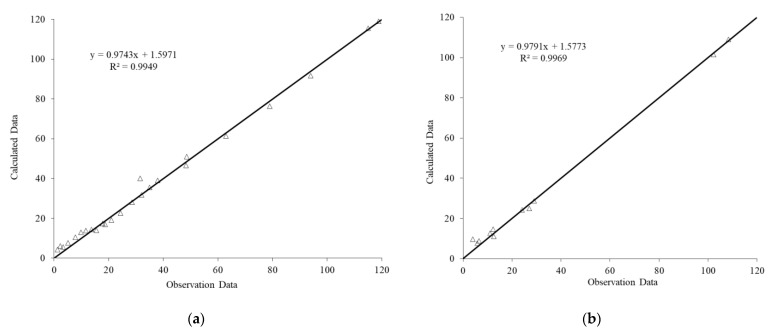
Predicted versus Experimental values of healing efficiency output using (**a**) training data, (**b**) testing data.

The results of the FOA-ANN models are shown in Figure 18 and Figure 19 for the degree of damage and healing efficiency output parameters, respectively. The results indicate that the FOA-ANN estimated a reliable result for the ratio of observational to computational values, R^2^, for both input parameters, indicating the proposed model’s high potential and accuracy.

Table 7 provide the final weights and biases for both hidden layers estimated by the FOA-ANN model. Using the values of the weights and biases between the different ANN layers, the two output parameters (degree of damage and healing efficiency) can be determined and predicted. Furthermore, these final weights and bias values can be used to design an epoxy-modified mortar with targeted mechanical properties and healing efficiency.

## 5. Conclusions 

This research investigated the application of epoxy resin polymer as a self-healing strategy for improving the mechanical and durability properties of cement-based mortar. Several mixes were designed with various ratios of the epoxy resin to OPC to assess the efficiency of the epoxy as the self-healing agent. The mechanical properties, ultrasonic pulse velocity (UPV), and SEM morphologies were recorded to determine the feasibility of implementing the epoxy resin as the self-healing agent in mortar manufacturing. In addition, by using the available experimental test database, an optimized ANN model combined with the firefly optimization algorithm (FOA-ANN) was developed to estimate the degree of damage and healing efficiency of mix designs. The following provides the main findings of this research.

(1) The final setting time and water absorption were significantly decreased by increasing the epoxy resin content in the modified mortar compared to a conventional specimen.
(2) The best mechanical properties were achieved by a specimen containing 10% epoxy resin, attributed to the presence of OH^−^ ions from the hydration of Ca(OH)_2_. However, the mechanical properties significantly decreased by increasing epoxy resin content from 10 to 15 and 20%. Such phenomenon can explain by the residual unhardened epoxy within the mortar matrix that may interrupt the hydration and polymerization processes.
(3) The SEM images indicated that the specimen prepared with 10% epoxy resin contain hydroxyl–ion–epoxy resin that led to the microstructure’s improvement, reduced the porosity, and providing high strength performance compared to the control specimen. In addition, in this specimen, the Ca(OH)_2_ was reacted with the unhardened epoxy resin, creating strong bonds between the hydroxyl ions and epoxy to increase the CS.
(4) The healing efficiency on CS and UPV is much higher for the specimen containing 10% epoxy resin than the conventional mortar. Furthermore, it was concluded that there is a direct relationship between artificial crack age and self-healing efficiency, where a higher healing efficiency was achieved at a younger crack age. Similarly, the degree of damage and healing efficiency were recorded 2.3 and 71.8%, respectively for the epoxy-modified specimen once the artificial cracks were generated at an early age.
(5) The ANN combined with the metaheuristic firefly algorithm provided satisfactorily results to estimate the degree of damage and healing efficiency in epoxy-modified specimens. Furthermore, the firefly algorithm optimization can also be used as a powerful tool in optimizing ANN weights. By using the optimized weight and bias of FOA-ANN, it is possible to design mixes with targeted degree of damage and healing efficiency depending on the particular environment.

## Figures and Tables

**Table 1:** Physical properties of epoxy resin.

Epoxy equivalent	184
Molecular weight	380
Flash point (°C)	264
Viscosity (cPs, 20 °C)	10,000
Density (g/cm^3^, 20 °C)	1.16

**Table 2:** Mix proportions of the self-healing mortars.

Mix	Cement (kg/m^3^)	Water (kg/m^3^)	River Sand (kg/m^3^)	Epoxy/Cement (%)
1 (control sample)	506	243	1518	0
2	506	243	1518	5
3	506	243	1518	10
4	506	243	1518	15
5	506	243	1518	20

**Table 3:** Degree of damage and Healing efficiency of epoxy-modified mortars.

Healing Efficiency of Modified Mortar Prepared with 10% Epoxy and 50% Pre-Loaded						
**A-Specimens pre-loaded at 1 month of curing age.**						
Concrete curing age, month	1	2	3	4	6	12
CS of non-loaded concrete, MPa	36.2	36.6	37.1	37.5	38.2	40.2
CS of pre-loaded concrete, MPa	18.1	23.8	24.8	26.9	29.5	35.1
Healing period, month	-	1	2	3	5	11
Degree of damage, %	21	11.6	9.4	8.9	8.1	2.3
Healing efficiency, %	-	29.3	32	41.4	51.9	71.8
**B-Specimens pre-loaded at 6 months of curing age.**						
Concrete curing age, month	6	7	8	9	12	18
CS of non-loaded concrete, MPa	38.2	38.9	39.2	39.8	40.2	41.8
CS of pre-loaded concrete, MPa	19.1	21.1	22.3	23.9	24.8	26.7
Healing period, month	-	1	2	3	5	11
Degree of damage, %	26	25.6	23.8	23.1	22.5	17.8
Healing efficiency, %	-	6.8	11.5	16.7	19.4	20.9

**Table 4:** Characteristics of studied input and output parameters.

Number	Parameter	Type	Unit	Max	Min	Average	STD
1	Epoxy	Input	kg/m^3^	50.6	0	37.95	22.22
2	Pre-Load at Age	Input	(Day)	360	28	149	138.65
3	Healing Duration	Input	(Month)	36	1	14	10.71
4	CS	Input	(MPa)	39.6	15.6	25.2	6.79
5	UPV	Input	(km/s)	4.26	2.93	3.45	0.37
6	Water Absorption	Input	(%)	14.8	5.5	9.57	3.08
7	Observation Degree of damage	Output	(%)	0.27	−0.08	0.16	0.09
8	Observation Healing efficiency	Output	(%)	118.78	0	34.04	34.7

**Table 5:** Statistical indices of ANN with 6-6-4-2 topology combined with FOA.

Training				Testing			
MSE	ME	MAE	RMSE	MSE	ME	MAE	RMSE
2.94	0.26	0.92	1.71	3.72	0.89	1.11	1.93

**Table 6:** Properties of FOA parameters.

Parameter	Value	Parameter	Value
Population size	100	Attraction coefficient base value	2
Mutation coefficient	0.25	Mutation coefficient damping ratio	0.99
Light absorption coefficient	1	M (exponent of distance term)	2

**Table 7:** Final weights and bias values of the optimum FA-ANN model 6-6-4-2.

**IW**						**b1**
−0.2226	0.6913	0.7234	0.4921	−0.2198	0.7664	0.3660
0.5876	0.2296	0.0543	−0.9068	−0.8038	0.7919	−0.4297
0.8156	0.8708	0.2050	−0.7444	−0.4160	−0.3075	0.9485
0.3177	−0.3422	−0.9205	−0.8923	0.5018	−0.0903	−0.4386
0.0142	−0.9034	−0.3105	0.9778	0.5866	0.3670	−0.0370
−0.7485	−0.0284	−0.7803	−0.2927	0.7430	−0.8874	−0.8910
**LW1**						**b2**
0.1391	−0.6483	−0.9244	−0.2580	0.3317	−0.2730	−0.5625
0.1451	−0.9574	0.9974	0.7265	−0.0867	−0.6183	0.5001
−0.0218	0.1769	−0.2386	0.5438	−0.2493	−0.0382	−0.8893
0.3847	−0.1255	−0.1702	0.4274	−0.7884	−0.6989	0.2426
**LW2**						**b3**
−0.4282	0.0805	0.6376	0.7041	-	-	0.0395
0.7643	0.4558	−0.4930	−0.4541	-	-	−0.3842

IW: Weights values for Input Layer, LW1: Weights values for First Hidden, LW2: Weights values for Second Hidden Layer, b1: Bias values for First Hidden Layer, b2: Bias values for Second Hidden Layer, b3: Bias values for Output Layer.

## Data Availability

Data sharing not applicable.
